# Supplement of levosimendan to epinephrine improves initial resuscitation outcomes from asphyxial cardiac arrest

**DOI:** 10.1186/s12871-017-0309-3

**Published:** 2017-02-02

**Authors:** Bingjing Wu, Yong G. Peng, Shishi Zhao, Nana Bao, Linmin Pan, Jiaojiao Dong, Xuzhong Xu, Quanguang Wang

**Affiliations:** 1 Department of Anesthesiology, The First Affiliated Hospital, Wenzhou Medical University, Wenzhou City, Zhejiang Province China; 2 Department of Anesthesiology, College of Medicine, University of Florida Shands Hospital, Gainesville, FL USA; 3 The First Affiliated Hospital, Wenzhou Medical University, South Baixiang Rd, Wenzhou City, Zhejiang Province 325000 China

**Keywords:** Asphyxia, Cardiac arrest, Epinephrine, Levosimendan, CPR

## Abstract

**Background:**

Levosimendan exerted favorable effects on the initial outcome in the treatment of ventricular fibrillation cardiac arrest. This study investigated the efficacy of levosimendan in the treatment of asphyxia-induced cardiac arrest in rats.

**Methods:**

Animals underwent asphyxial cardiac arrest/cardiopulmonary resuscitation, randomized to three treatment groups: epinephrine (10 μg/kg) supplemented with levosimendan (bolus 12 μg/kg and infusion for 1 h, EL group); epinephrine only (10 μg/kg, E group), or levosimendan only (bolus 12 μg/kg and infusion for 1 h, L group). The resuscitation success rate, wet-to-dry ratio of lung, and rate of alveolar and blood gas analysis were recorded.

**Results:**

10 rats in the EL group, 8 in the E group, and 2 in the L group showed an initial return of spontaneous circulation (*P* < 0.001); among them, 10, 4, and 2 rats survived at the end of a 60-min observation period from each group, respectively (*P* = 0.001). The coronary perfusion pressure in the EL group was higher than that of either the E or L group (*P* < 0.05). The lung wet-to-dry weight ratio and rate of damaged alveoli were lower in the EL group than the E group (*P* < 0.05).

**Conclusions:**

In the early stage of resuscitation for asphyxia-induced cardiac arrest in rats, levosimendan supplemented with epinephrine can significantly increase coronary perfusion pressure, reduce lung injury, and ultimately enhance the survival rate.

**Electronic supplementary material:**

The online version of this article (doi:10.1186/s12871-017-0309-3) contains supplementary material, which is available to authorized users.

## Background

Cardiac arrest (CA) is the most devastating adverse cardiac event, and it causes approximately 325,000 deaths each year in the United States [1]. Asphyxia, a result from loss of the airway, is a rare but significant cause of serious complications, and can lead to an asphyxia-induced CA. Epinephrine is a standard of medication in resuscitation of CA and it has been incorporated into the American Heart Association guidelines for cardio-pulmonary resuscitation since 1973. It exhibits potent vasoconstriction effect, increases coronary perfusion pressure (CPP), and facilitates return of spontaneous circulation (ROSC) [2, 3] However, the effects of sole epinephrine in the setting of CA have always been questioned because it increases myocardial oxygen demand through its beta adrenergic receptors [4], which cause post-resuscitation myocardial dysfunction [5]. It also exerts acute, adverse effects on pulmonary oxygen exchange, which then leads to severe pulmonary edema and acidosis [6]. These unwarranted side effects have a negative impact on long-term survival. Therefore, the American Heart Association guidelines have now discouraged large doses of epinephrine for adult advanced cardiovascular life support during resuscitation [7]. In clinical practice, however, epinephrine is still the preferred agent during cardiopulmonary resuscitation (CPR).

Levosimendan is a unique inodilator that exerts inotropic effects principally via binding to the Ca^2+^-saturated troponin C of the myocardial thin filament [8]. Unlike classic inotropic agents, it has inotropic action without increasing myocardial oxygen consumption during infusion in congestive heart failure [9]. Furthermore, levosimendan also has vasodilatory effects mediated by the opening of ATP-sensitive potassium channels in the sarcolemmal membrane of vascular smooth muscle cells [10], which decreases the central venous pressure and the systolic and diastolic pressures of the right atrium [11].

There are conflicting reports in the literature regarding the efficacy of levosimendan in the treatment of CA. Kelm et al. [12] reported that levosimendan combined with vasopressin administration during CPR resulted in increased cerebral blood flow and improved neurological outcome, but could not facilitate ROSC in a rat model of asphyctic CA. The recent research reported by Kosmidou et al. [13] showed that levosimendan combined with epinephrine only improved 24-h neurological outcome, but there was no evidence of improvement of initial resuscitation success and the final survival rate in a swine model of asphyctic CA. However, Koudouna et al. [14] reported that a combination of epinephrine and levosimendan in a swine model of ventricular fibrillation CA significantly improved CPP and initial resuscitation success. Thus, it is not clear whether levosimendan could play a beneficial role in the process of resuscitation; additional studies are necessary for providing further evidence to confirm the benefit of levosimendan during resuscitation with different animal CA models.

We hypothesized that levosimendan combined with epinephrine can improve post-resuscitation outcomes and survival rates in the treatment of asphyxia-induced CA, and designed a double-blind, prospective, randomized study using a rat model after asphyctic CA. The primary end point assessed was the survival rate, and the secondary end points were the rate of ROSC, the lung wet-to-dry weight ratio, and hemodynamic parameters.

## Methods

### Experimental animals and groups

All studies were approved by the Ethics Committee of the Wenzhou Medical University (Wenzhou, China). Healthy Sprague-Dawley male rats, 7 to 8 weeks old, 300 to 350 gin weight, were divided randomly into three experimental groups (10rats/group) randomly: the epinephrine (Jinyao Amino Acid Co., Ltd., Tianjin, China) and levosimendan (Qilu pharmaceutical Co., Ltd., Shandong, China) treatment group (EL), the epinephrine (10 μg/kg in 2.5 ml of volume) combined with levosimendan [bolus during CPR (12 μg/kg in 1.2 ml of volume) and infusion for 1 h (0.3 μg/kg/min in 0.03 ml of volume)], and the epinephrine-only treatment group (E), epinephrine (10 μg/kg in 2.5 ml of volume) administered with saline 0.9% [bolus during CPR and infusion for 1 h (equivalent fluid volume)] or levosimendan-only treatment group (L), saline 0.9% (equivalent fluid volume bolus) administered with levosimendan [bolus 12 μg/kg in 1.2 ml of volume and infusion for 1 h (0.3 μg/kg/min in 0.03 ml of volume)].

### Animal preparation

All rats were fasted for 12 h before the experiment began and were given access to water ad libitum. They were anesthetized with an intraperitoneal injection of urethane (20%, 200 mg/kg). A tracheal intubation was performed via tracheotomy and rats were connected to the rodent volume-controlled ventilator (tidal volume, 8 ml/kg; FiO_2_,1.0; respiratory rate, 75–80 breaths/min; inspiratory/expiratory ratio, 2:3; HX-300; TME Technology Co., Ltd., Chengdu, China) [15, 16]. The right femoral artery was cannulated for the sampling of blood and continuous arterial pressure monitoring. Intravenous drug administration took place through drug right femoral vein cannulation. A third catheter was inserted into the left jugular vein and then advanced into the right atrium for measuring the right atrial pressure. Electrocardiography, using three subcutaneous needle electrodes, continuously recorded arterial and right atrial pressure throughout the duration of the experiments with a MedLab data archiving and retrieval system using U/4C051 (Nanjing Medease Science and Technology Co., Ltd., Jiangsu, China). All animals were stabilized for 15 min after completion of invasive procedures. Thereafter, CPP, mean arterial pressure (MAP), heart rate (HR), and rate-pressure product (RPP) were recorded. CPP was calculated as the difference between decompression diastolic aortic and time-coincident right atrial pressure and was measured at the end of each minute of precordial compression [14]. Arterial blood gases (ABGs) were also collected [17].

### Experimental model

The asphyxia-induced CA model was quoted from the experiment performed by McCaul et al. [18]. After a stabilization periodof15 min, CA was achieved by stopping mechanical ventilation, which resulted in CA after approximately 3 min. CA was indicated by a decrease in MAP below 10 mmHg. All rats in each group were led to CA.

### Resuscitation protocol

Resuscitation began 1 min after onset of CA and was established by starting the ventilator (FiO_2_ 1.0, V_T_ 8 ml/kg, rate 75–80 min^−1^), beginning external chest compressions (approximately 300 min^−1^ at a depth of 1 cm), and through intravenous administration of medication. Effective resuscitation (ROSC) was defined as a native RPP more than 20% of baseline value for 1 min [15, 19]. Chest compressions continued until spontaneous circulation returned or an elapsed time of 60 min without successful resuscitation; no ventilation changes were made before or after resuscitation. We used an adjustable infrared sensor maintain body temperature at 37 °C to 39 °C, and we preheated all intravenous solutions to 37 °C before infusion. At the end of the 60-min resuscitation period, the animals were killed by anesthetic overdose, and the excised lung samples were taken for subsequent analysis.

### Measurements

We dynamically documented the values of SBP, MAP, and HR in all three groups and calculated the RPP and CPP of the survivors during the 60-min resuscitation period. In addition, we recorded the time to CA, the time to first heartbeat, and the time to ROSC. ROSC and rats surviving at 60 min were also recorded. The rate of successful resuscitation and survival at 60 min was calculated (rate of ROSC = number of rats displaying ROSC/total number of rats; survival at 60 min = number of rats survived at 60 min/total number of rats). Finally, arterial blood gas analyses were performed.

### The wet-to-dry ratio of the lung and pathology

The animals were sacrificed after CPR. The lobe of the left lung was weighed and dried, and we calculated the lung wet-to-dry weight ratio. We fixed the middle lobe of the right lungin10% formalin fixative, created paraffin-embedded slices, and used hematoxylin and eosin stain for observation [15]. Fifty views (400×) were selected randomly in each sample by using light microscopy. In every view, the total number of pulmonary alveoli and the number damaged were counted. We then calculated the ratio of damaged lung alveoli to the total number of lung alveoli. We defined the alveolus as injured when more than two inflammatory cells or two red blood cells were evident in one pulmonary alveolus [15, 20].

### Statistical analysis

Based on our pre-test, we used the Power Sample Size (PASS11.0) software program for power analysis and compared the survival rates among the experimental groups. In our preliminary study, 18 rats were used, with 6 rats in each group. There were 6, 1, and 1 rat in groups EL, E, and L that survived to 60 min, respectively. The survival rate was 100, 16.7, and 16.7%, respectively. We set the power at 0.8; the significance criterion was 0.017. As a result, we needed 8 rats per group to achieve statistical significance. To account for potential attrition, we enrolled 10 rats per group.

All data were performed using SPSS for 17.0 for Windows. All data generated or analysed during this study are included in this published article (Additional file 1). The measurement data were presented as means ± standard deviation (SD), and in the case of categorical variables, frequencies were used. We used the Shapiro-Wilk test to analyze normal distribution. Differences of baseline parameters, time to CA, time to first heartbeat, time to ROSC, blood gas parameters, lung wet-to-dry ratio, and the ratio of damaged alveoli in the three groups were compared by one-way analysis of variance. We used the least significant difference test for the data that had homogeneity of variance, and the Dunnett’s T3 test was used for the data that exhibited heterogeneity of variance. Differences of hemodynamic parameters in animals were analyzed by two-way, repeated-measures analysis of variance, and Bonferroni post hoc analysis when significance was achieved. *P* value < 0.05 was considered significant. Fisher probabilities in a 3 × 2 table were used for analyzing success resuscitation rates and survival rates, and *P* value < 0.017 was considered significant in post hoc multiple comparisons.

## Results

### Baseline values

There were no differences observed in weight, baseline hemodynamic metrics, or baseline blood gas values among the groups (Table 1).

**Table 1:** Baseline Values of Weight, Hemodynamic Metrics, and Blood Gas Values for group EL, group E and group L

	EL group (*n* = 10)	E group (*n* = 10)	L group (*n* = 10)	*P* value
Weight, g	320 ± 16	326 ± 12	332 ± 13	0.174
SBP, mmHg	113 ± 11	108 ± 7	114 ± 9	0.310
RPP, mmHg · beat · min^−1^	50886 ± 6330	47889 ± 3750	47997 ± 5075	0.352
CPP, mmHg	72 ± 10	69 ± 9	73 ± 7	0.695
PH	7.39 ± 0.03	7.39 ± 0.04	7.37 ± 0.04	0.248
PaO_2_, mmHg	369 ± 32	355 ± 40	389 ± 62	0.290
HCO_3_^−^, mmol/L	22.6 ± 2.6	22.2 ± 2.3	24.2 ± 2.4	0.176
BE, mmol/L	−2.3 ± 3.1	−2.7 ± 2.1	−1.5 ± 2.1	0.550
Lactate, mmol/L	1.1 ± 0.5	1.2 ± 0.4	1.5 ± 0.3	0.074

Normally distributed data were given as mean ± SD. No statistical difference among the 3 groups. SBP indicates systolic blood pressure. RPP indicates rate-pressure product (systolic blood pressure × heart rate). CPP indicates coronary perfusion pressure. BE indicates base excess

### Resuscitation outcomes

**Fig. 1:**
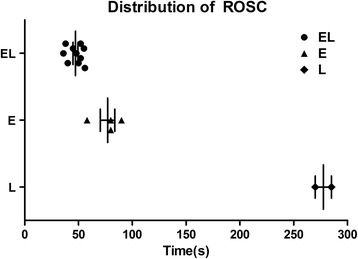
A scatter plot of the time to ROSC for rats that survived to 60 min. The numbers of rats that survived to 60 min in EL, E, and L groups were 10, 4, 2, respectively. The times to ROSC in the EL, E, and L groups were 47 ± 7 s, 77 ± 14 s, 278 ± 11 s, respectively. The times to ROSC in the three groups displayed statistical differences (*P* < 0.001, EL vs. E, *P* < 0.001; EL vs. L, *P* < 0.001; E vs. L, *P* < 0.001). n = 10 for the EL group, *n* = 4 for the E group, *n* = 2 for the L group. ROSC: return of spontaneous circulation

All animas in each group experienced CA for approximately 3 min, and no significant difference was found among the three groups in the time to CA (*P* = 0.986; Table 2). The numbers of animals with ROSC in the EL, E, and L groups were 10, 8, and 2, respectively; the three groups displayed different ROSC rates (10 rats in each group; *P* < 0.001; Table 2). The numbers of animals surviving to 60 min were 10, 4, and 2, in the EL, E, and L groups, respectively. The EL, E, and L groups thus displayed a difference in rates of survival (*P* = 0.001; Table 2). The groups also demonstrated differences in the time to return of pulse (*P* < 0.001; Table 2) and the time to ROSC (*P* = 0.001; Table 2). The time to ROSC of the survived rats, and its distribution in the three groups, is presented in Fig. 1.

**Table 2:** Resuscitation Outcomes for EL, E and L Groups

	EL group (*n* = 10)	E group (*n* = 10)	L group (*n* = 10)	*P* value
Rate of ROSC, n (%)	10 (100%)^##^	8 (80%)	2 (20%)^*^	<0.001
Survival rate, n (%)	10 (100%)^*##^	4 (40%)	2 (20%)	0.001
Time to CA, s	167 ± 28	167 ± 26	165 ± 19	0.986
Time to return of pulse, s	39 ± 7^##^	44 ± 10	99 ± 19^**^	< 0.001
Time to ROSC, s	47 ± 7^**#^	81 ± 22	277 ± 10^**^	0.001

Normal distributed data are given as mean ± SD. The EL, E and L groups displayed differences in the rate of ROSC (*P* < 0.001; EL vs. E, *P* =0.474; EL vs. L, *P* = 0.001; E vs. L, *P* =0.023). The EL, E and L groups displayed difference in rates of survival (*P =* 0.001; EL vs. E, *P* = 0.011; EL vs. L, *P* = 0.001; E vs. L, *P* =0.628). There were no differences among the three groups in the time to CA (*P* > 0.05). The EL, E and L groups displayed differences in the time to return of pulse (*P* < 0.001; EL vs. E, *P* =0.354; EL vs. L, *P* < 0.001; E vs. L, *P* < 0.001). The EL, E, and L groups displayed differences in the time to ROSC (*P* = 0.001; EL vs. E, *P* = 0.008; EL vs. L, *P* = 0.021; E vs. L, *P* < 0.001), ^*^ *P* < 0.05, ^**^ *P* < 0.01, versus group E;^#^ *P* < 0.05, ^##^ *P* < 0.01, versus group L. ROSC indicates return of spontaneous circulation. CA indicates cardiac arrest

### Hemodynamic measures

**Fig. 2:**
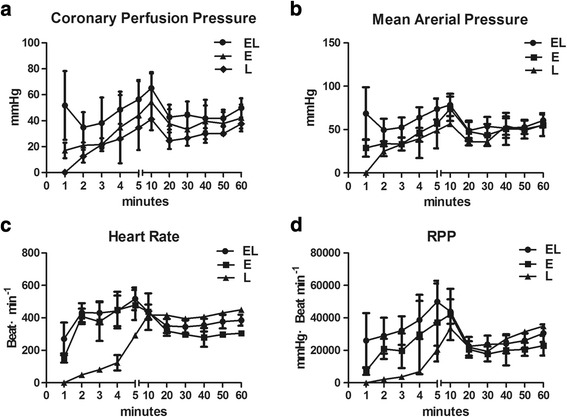
Hemodynamic parameters for rats that survived to 60 min. CPP (**a**), MAP (**b**), HR (**c**), and RPP (**d**) versus time for these survival rats during the 60-min observation period are shown. The data are presented as means and SD. Significant differences are demonstrated in the values of CPP, MAP, HR, and RPP among the EL, E, and L groups (*P* < 0.05). On considering the importance of the early phase for successful resuscitation, further comparisons in the first 3 min were performed. CPP and MAP, but not RPP or HR in the EL group was higher than that of the E or L group within the first 3 min (*P* < 0.05).*n* = 10 for the EL group, *n* = 4 for the E group, *n* = 2 for the L group. CPP = coronary perfusion pressure; MAP = mean arterial pressure; HR = heart rate. RPP = rate-pressure product(systolic blood pressure × heart rate)

Hemodynamic values such as CPP, MAP, HR, and RPP for the three groups are presented in Fig. 2. Significant differences were seen in CPP, MAP, HR, and RPP among the EL, E, and L groups (*P* < 0.05). Further comparisons in the first 3 min, e.g., the CPP and MAP, but not the RPP or HR, in the EL group were higher than that in the E or L group (*P* < 0.05).

### Lung wet-to-dry weight ratio

**Fig. 3:**
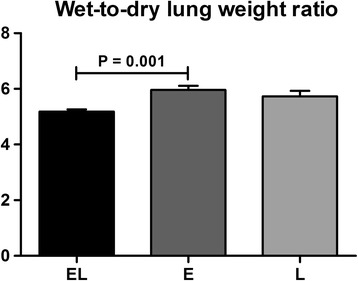
Lung wet-to-dry ratio. Normal distributed data are given as mean ± SD. The EL, E, and L groups displayed statistic differences in wet-to-dry lung weight ratios (5.1 ± 0.3, 6.0 ± 0.4, and 5.7 ± 0.6, respectively; *P* = 0.005). The ratio in the EL group was significantly lower than that of the E group, *P* = 0.001

The EL, E, and L groups showed differences in lung wet-to-dry weight ratios (5.1 ± 0.3, 6.0 ± 0.4, and 5.7 ± 0.6, respectively; *P* = 0.005). The ratio in the EL group was significantly lower than that of the E group (*P* = 0.001; Fig. 3).

### Lung histological examination

**Fig. 4:**
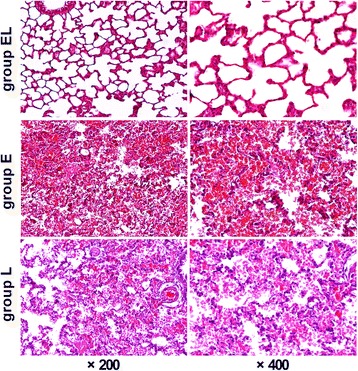
The samples were taken from an EL, E, and L group rat’s right middle lobe, and the view was under light microscopy. For the rats in the EL group, the alveolar structures are normal and there is no leukocyte or erythrocyte accumulation seen in the alveoli. In the E group, most of the alveoli are destroyed, and their structures are significantly altered, with numerous erythrocytes evident accompanying the damaged alveolar framework. In the L group, some alveoli are identified, and there are numerous erythrocytes observed in the alveoli. The EL, E, and L groups displayed statistical differences in the rate of damaged alveoli (0.15 ± 0.07, 0.74 ± 0.08, and 0.53 ± 0.31, respectively; *P* = 0.002). The rate of damaged alveoli in the EL group was significantly lower than that in the E group, *P* < 0.001. Magnification: 200× (left panel), 400× (*right panel*)

In the EL group, there was no evidence of alveolar structural damage, edema, or hemorrhage. In the E group, there were numerous erythrocytes in the alveolar field, accompanied by altered alveolar structures. In the L group, some alveoli could be identified, and there were numerous erythrocytes observed in the injured alveoli (Fig. 4). The EL, E, and L groups showed differences in the rate of injured alveoli (0.15 ± 0.07, 0.74 ± 0.08, 0.53 ± 0.31, respectively; *P* = 0.002). The rate of damaged alveoli in the EL group was significantly lower than that of the E group (*P* < 0.001).

### Blood gas analysis

Arterial blood gas parameters at 60 min are shown in Table 3. The EL, E, and L groups displayed significant differences in blood gas parameters (*P* < 0.05). Specifically, the PH, PaO_2_, HCO_3_^-,^ and base excess (BE) values of the EL group were significantly higher than that of the E group (PH: EL vs. E, *P* = 0.021; PaO_2_: EL vs. E, *P* < 0.001; HCO_3_^−^: EL vs. E, *P* = 0.004; BE: EL vs. E, *P* = 0.004). In addition, the PH values and BE in the EL group were significantly higher than that of the L group (PH: EL vs. L, *P* = 0.024; BE: EL vs. L, *P* = 0.01). The blood lactate value in the EL group was significantly lower than that of the E or L group (EL vs. E, *P* = 0.018, EL vs. L, *P* = 0.001; Table 3).

**Table 3:** Arterial Blood Gas Parameters at 60 min

	EL group (*n* = 10)	E group (*n* = 10)	L group (*n* = 10)	*P* value
PH	7.32 ± 0.02^*#^	7.06 ± 0.24	7.14 ± 0.18	0.01
PaO_2_, mmHg	173 ± 33^**^	91 ± 27	93 ± 85	0.008
HCO_3_^−^, mmol/L	21.6 ± 1.6^**^	14.3 ± 5.1	19.0 ± 4.0	0.001
BE, mmol/L	−4.6 ± 1.7^**#^	−16.5 ± 8.3	−10.1 ± 4.5	0.001
Lactate, mmol/L	1.1 ± 0.5^*##^	8.0 ± 6.2	8.7 ± 4.0	0.002

Normal distributed data are given as mean ± SD. The EL, E and L groups demonstrated differences in blood gas values. The pH, PaO_2_, HCO_3_^−^ and BE values in the EL group were higher than that in the E group (PH: EL vs. E, *P* = 0.021; PaO_2_: EL vs. E, *P* < 0.001; HCO_3_^−^: EL vs. E, *P* = 0.004; BE: EL vs. E, *P* = 0.004). The PH and BE values in the EL group were higher than that in the L group (PH: EL vs. L, *P* = 0.024; BE: EL vs. L, *P* = 0.01). The blood lactate value in the EL group were lower than that of the E or L group (EL vs. E, *P* = 0.018; EL vs. L, *P* = 0.001). There were no differences between EL group and L group in the PaO_2_ and HCO_3_^−^ values (*P* > 0.05). ^*^ *P* < 0.05, **P < 0.01, versus group E; ^#^ *P* < 0.05, ^##^ *P* < 0.01, versus group L. BE indicates base excess

## Discussion

In our study of resuscitation on asphyxia-induced CA, we demonstrated that CPP significantly improves when rats received epinephrine therapy supplemented with levosimendan. The EL group also exhibited a significant reduction in lung injury and acidosis, excellent oxygen partial pressure, and an improved survival rate compared with rats that received epinephrine or levosimendan alone.

CPP, calculated as aortic pressure minus right atrial pressure during the diastolic phase of CPR, is strongly associated with resuscitation outcomes [17, 21]. Friess et al. reported that maintaining CPP > 20 mmHg is the primary determinant for ROSC and survival from CA [22]. Levosimendan can decrease the central venous pressure and the systolic and diastolic pressures of the right atrium [11]. Epinephrine can constrict the peripheral blood vessels and increase aortic pressure. Concomitant therapy with epinephrine and levosimendan has a synergistic effect on elevating CPP. In our study, CPP in the epinephrine supplemented with levosimendan group increased more significantly than the other two groups within 3 min, with an average CPP > 20 mmHg. It may be this synergistic effect that leads to all rats exhibiting ROSC in the EL group, suggesting that maximizing CPP quickly and efficiently is the key component to improving CPR outcomes. Levosimendan alone can not enhance CPP during CPR because of its peripheral vasodilatory effect. The result of our study was that CPP and the resuscitation rate of the levosimendan group was the lowest among the three groups.

Myocardium contraction weakened when heart failure, correlated with the decrease in extracellular Ca^2+^ inflow, reduced the sarcoplasmic reticulum uptake of release of Ca^2+^ and hindered the binding between troponin and Ca^2+^. Levosimendan is a selective systolic calcium sensitizer mainly used clinically for the treatment of acute heart failure. When sudden CA occurs, synthesis of ATP is blocked and myocardial cell cannot pump redundant Ca^2+^ to the extracellular space, thereby inhibiting excitation contraction coupling. Myocardial cytoclasis then occurs due to an overload of calcium. If CPR is implemented immediately, myocardial perfusion would maintain calcium ion at 25 to 30% of normal levels. Epinephrine is added at the same time to constrict the blood vessels to enhance CPP. In addition, levosimendan has a direct vasodilatory effect on coronary arteries and enhances coronary blood flow [23, 24]. In this case, myocardial cells decompose glucose to obtain the minimal requirement of ATP to continue the mechanism of calcium-triggered calcium release. If so, levosimendan could exert the function of sensitization to strengthen the contractility of myocardial cells.

Krishnamoorthy et al. [6] reported that intravenous injection of epinephrine in healthy adult rats induced rapid deterioration of pulmonary oxygen exchange, but the effects were blunted by α-adrenergic receptor blockade. Lindberg et al. [25] showed that epinephrine caused peripheral vasoconstriction through excitation of alpha receptors, which increased cardiac afterload, and could further increase left atrial and pulmonary vein pressure. In addition, the Morelli study suggested that levosimendan could expand the peripheral vascular bed and decrease the cardiac afterload in patients who suffer from acute respiratory distress syndrome [26]. Bracken et al. [27] also demonstrated that levosimendan reduced the pressure of cat pulmonary arteries. In the present experiment, rats receiving epinephrine supplemented with levosimendan showed less lung injury and acidosis after resuscitation. This result suggests that the vasodilatory effects of levosimendan could partly offset the epinephrine-induced side effects. The complementary effect of these two medications could xhave possible beneficial CPR outcomes CPR outcomes.

Many α-agonist agents have been shown to increase CPP. However, no placebo-controlled study has shown that the use of any vasoconstrictor during CA can increase survival rates [14]. Kelm et al. [12] reported that levosimendan combined with vasopressin administration during CPR resulted in increased cerebral blood flow and improved neurological outcomes. However, their study failed to demonstrate that the combination of the above two medications can facilitate ROSC in a rat model of asphyctic CA. Kosmidou et al. [13] showed that 12 μg/kg levosimendan combined with 20 μg/kg epinephrine only improved 24-h neurological outcomes in a swine model of asphyctic CA. However, Koudouna et al. [14] reported that the same dose of levosimendan combined with epinephrine significantly improved CPP and initial resuscitation success in a swine model of ventricular fibrillation CA. In our study, 10 μg/kg epinephrine combined with 12 μg/kg levosimendan significantly improved survival rates in a rat model of asphyctic CA. The difference of species and dosage may result in encouraging results our study has observed. Nevertheless, the ideal dosage of each medication to produce the best effect is still not clear, which requires additional studies on the dosage-response relationship of epinephrine and levosimendan on CPR.

It should be recognized that this study has limitations. Our study focused exclusively on short-term survival results and does not address neurological outcomes and long-term survival outcomes, which are the end point of cardiopulmonary cerebral resuscitation in clinical practice. We will explore these parameters in future experiments.

## Conclusion

As a calcium-sensitizer agent, levosimendan improved initial resuscitation outcomes in asphyxia-induced CA when administered with epinephrine during CPR. These effects may contribute to increased coronary perfusion flow and reduced lung injury and acidosis. Our study has suggested that levosimendan can be selected as a promising alternative supplement agent to epinephrine during CPR for asphyxia-induced CA.

## Additional file

Additional file 1: All data generated or analysed during this study. (ZIP 67 kb)

## Abbreviations

BE: Base excess
CA: Cardiac arrest
CPP: Coronary perfusion pressure
CPR: Cardiopulmonary resuscitation
HR: Heart rate
MAP: Mean arterial pressure
ROSC: Return of spontaneous circulation
RPP: Rate-pressure product
